# New Insights into Pathomechanisms and Treatment Possibilities for Lung Silicosis

**DOI:** 10.3390/ijms22084162

**Published:** 2021-04-17

**Authors:** Jana Adamcakova, Daniela Mokra

**Affiliations:** Department of Physiology, Jessenius Faculty of Medicine in Martin, Comenius University in Bratislava, SK-03601 Martin, Slovakia; jana.adamcakova@gmail.com

**Keywords:** silica, silicosis, inflammation, oxidative stress, lung fibrosis

## Abstract

Inhalation of silica particles is an environmental and occupational cause of silicosis, a type of pneumoconiosis. Development of the lung silicosis is a unique process in which the vicious cycle of ingestion of inhaled silica particles by alveolar macrophages and their release triggers inflammation, generation of nodular lesions, and irreversible fibrosis. The pathophysiology of silicosis is complex, and interactions between the pathomechanisms have not been completely understood. However, elucidation of silica-induced inflammation cascades and inflammation-fibrosis relations has uncovered several novel possibilities of therapeutic targeting. This article reviews new information on the pathophysiology of silicosis and points out several promising treatment approaches targeting silicosis-related pathways.

## 1. Silica and Silicosis

Silica (silicon dioxide, SiO_2_, or quartz) naturally occurs in the earth´s crust and can be released in mining, sandblasting, quarrying, or fabrication of artificial stone [1,2]. Silica exists in several crystalline and amorphous forms with diverse physio-chemical properties. Among them, crystalline silica with its polymerized tetrahedral framework has the highest pathogenicity [2,3]. Professional exposure to crystalline silica may lead to: (**1**) chronic silicosis as a result of more than 10 years of low-moderate exposure dose; (**2**) accelerated silicosis, which occurs within 10 years of moderate-high exposure dose; or (**3**) acute silicosis (or silicoproteinosis), which develops within 5 years of very high exposure dose [1,2]. Chronic silicosis is recognized in two forms. Simple (or nodular) chronic silicosis is characterized by a development of small (<1 cm in size) and hard nodules in the upper lung lobes. These patients may be asymptomatic or may have dry cough or exertional dyspnoea [4]. The situation may become complicated when the silicotic nodules fuse to form conglomerate masses (>1 cm in size), which is characteristic of the second form of chronic silicosis, progressive massive fibrosis. Clinical signs may be identical to simple silicosis or may be more serious, with the development of central cavitation increasing the risk of mycobacterial infection, enlarged hilar or mediastinal lymphadenopathy, pleural thickening, higher risk of spontaneous pneumothorax, and weight loss [5]. Accelerated silicosis may initially have a similar pattern to simple chronic silicosis, but later the development of nodules and masses intensifies [6]. Acute silicosis may occur within several weeks or less than 5 years of high-intensity silica exposure, which causes dyspnoea and a cough and rapidly progresses to respiratory failure. Acute silicosis is associated with hypertrophy of alveolar cells type II and production of excessive amounts of proteinaceous material, including surfactant proteins [3].

## 2. Pathomechanisms of Lung Silicosis

**Figure 1:**
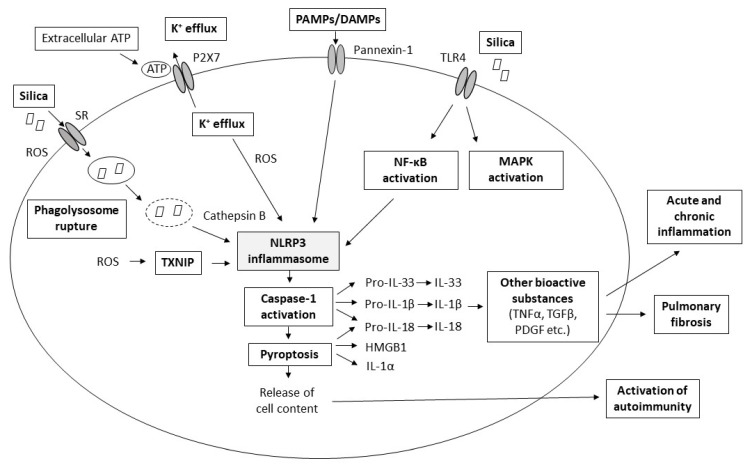
Scheme of major proposed pathomechanisms of pulmonary silicosis. More details about the individual pathomechanisms are provided in the following text. Abbreviations: ATP: adenosine triphosphate, DAMPs: danger-associated molecular patterns, HMGB1: high mobility group box 1 protein, IL: interleukin, K^+^: potassium cations, MAPK: mitogen-activated protein kinase, NF-κB: nuclear factor kappa B, PAMPs: pathogen-associated molecular patterns, PDGF: platelet-derived growth factor, ROS: reactive oxygen species, SR: scavenger receptors, TGFβ: transforming growth factor beta, TLR: Toll-like receptor, TXNIP: thioredoxin-interacting protein.

The background of pathological changes in silica-injured lungs is complex and not completely understood. Silica-induced lung injury presumably results from the combined action of several interacting pathomechanisms, such as the direct cytotoxic effect of silica on macrophages, activation of macrophage surface receptors, lysosomal rupture, production of reactive oxygen species (ROS), activation of inflammasome, production of cytokines and chemokines, cell apoptosis/pyroptosis, and lung fibrosis [1,7,8,9] (Figure 1).

### 2.1. Recognition of Silica by Macrophage Scavenger Receptors

Silica particles are recognized by receptors localized on the surface of alveolar macrophages. Among all classes of scavenger receptors (SR), transmembrane proteins SR-AI, SR-AII and macrophage receptors with a collagenous structure (MARCO) are most associated with silica binding [10]. SR may play a role in initiation of apoptosis of alveolar macrophages [11]. However, other pattern recognition receptors, e.g., the toll-like receptor (TLR)4 or type 3 complement receptor, may also participate in silica binding [7].

### 2.2. Direct Cytotoxic Effect of Silica Particles on Macrophages

Respirable silica particles (<10 μm), which pass through a mucociliary defence mechanism, may reach distal lung compartments where they initiate a cascade of actions, leading to the development of lung silicosis [12]. In the terminal airways and alveoli, the inhaled silica is engulfed by alveolar macrophages, which are primarily responsible for clearing the lung from debris [7]. However, silica is extremely toxic for macrophages. If alveolar macrophages survive contact with silica, they can migrate out of the lung, or they can move to the lung interstitium, where they change into activated interstitial macrophages, important for progression of silica-induced lung injury [13,14]. As discussed in a review by Hamilton et al. [7], the cytotoxicity of silica particles may be explained by several hypotheses. Crystalline silica (quartz, cristoballite, and some forms of tridymite) are inherently piezoelectric, i.e., they generate opposite electric charges on opposite sides of the physical structure during the application of pressure. These piezoelectric properties of the crystals, particularly of those freshly fractured, trigger the generation of ROS. In addition, silanol (SiOH) groups present on the surface of silica, although not free radicals, may form hydrogen bonds with oxygen and nitrogen groups in the cell membranes. Thus, the initial toxicity to macrophages and silica-induced production of ROS seem to be the critical mechanisms of initiation and progression of inflammation and fibrosis [3,15,16].

Before inhaled silica is engulfed by alveolar macrophages, the surface of the silica is coated by components of pulmonary surfactant, likely to protect from silica-induced lung injury. It is presumed that alveolar macrophages in response to silica stimulation enhance type II cells to produce more surfactant [7,17]. However, although surfactant is increasingly produced after silica inhalation, its effect is only temporary because of early destruction by enzymes [18,19]. On the other hand, high production of surfactant may complicate the situation as surfactant phospholipids and proteins undergo oxidative modifications in contact with crystalline silica, and uncontrolled surfactant overproduction may lead to acute silicoproteinosis [7,20].

### 2.3. Production of Reactive Oxygen and Nitrogen Species

There are several sources of ROS and reactive nitrogen species (RNS) associated with interaction of silica with macrophages. In vitro and in vivo studies confirmed that freshly fractured silica particles are more toxic than aged ones [21,22], while even more cytotoxic is the silica contaminated with iron producing more hydroxyl radicals [23]. Particle-derived production of ROS results from a homolytic cleavage of silicon-oxygen bonds to Si^•^ and SiO^•^, or from a heterolytic cleavage generating Si^+^ and SiO^+^ [24]. These by-products may react with oxygen, and the freshly cut silica may also react with carbon dioxide or water to form several other ROS [7]. Additional free radicals including superoxide anion, hydroxyl radicals, and hydrogen peroxide are generated, and nitric oxide synthase (NOS) is increased in activated phagocytic cells in response to silica [25]. Silica exposure can also stimulate the activities of antioxidants, as demonstrated in the rat lung where silica inhalation increased manganese-containing superoxide dismutase (MnSOD) mRNA and glutathione peroxidase (GPx) mRNA levels [26].

Besides direct cytotoxicity, free radicals act as important initiators of a wide cascade of cellular responses, including mitogen-activated protein kinase (MAPK) phosphorylation, activation of transcription factors nuclear factor (NF)-κB and activator protein (AP)-1, and activation of inflammasome [8].

### 2.4. Rupture of Lysosomes

After recognition of silica by alveolar macrophages, they engulf the silica particles. The internalized silica is entrapped by lysosomes, where a low pH-activated variety of enzymes (phosphatases, proteases, nucleases, and enzymes hydrolyzing polysaccharides or lipids) is prepared to digest the particle. Enzymes also despoil the protective surfactant coating on the surface of silica particles [27]. However, the silica particle cannot be broken down by the enzymes, which results in the loss of lysosomal membrane integrity and the release of lysosomal enzymes, including protease cathepsin B. Cathepsin B activates caspase-1 and cell apoptosis as well as inflammasome [28,29]. Freed silica particles can be bound and internalized by other macrophages, creating a vitious cycle of the toxic effects of silica on alveolar macrophages [7]. ROS generated in phagocytosis and cell contents released due to cell apoptosis act as potent activators of various processes including the activation of inflammasome [8].

### 2.5. Activation of Inflammasome

As recently reviewed in several excellent articles [8,9,27,30,31], silica-induced activation of inflammasome is a fundamental pathway leading to lung injury. Here, we present only a short overview of inflammasome-mediated processes, which is essential for understanding the potential therapeutical targets provided in the following subsections of this article.

Besides surface membrane-bound receptors such as TLRs, which survey the extracellular environment, the innate immune system also contains a special system of intracellular receptors, e.g., the nucleotide-binding and oligomerization domain (NOD)-like receptors (NLRs) [32]. In silica-induced lung inflammation and injury, NLRP3 inflammasome is of fundamental importance. NLRP3 inflammasome is an intracellular complex structure that contains a C-terminal leucine-rich repeat (LRR) for ligand recognition, a central NBD (NACHT) domain for an oligomerization, and a N-terminal pyrin domain (PYD) for a signal transduction [9]. This NLRP3 protein binds to an adaptor protein (apoptosis-associated speck protein) with a caspase activation and recruitment domain (ASC), and the caspase-1. NLRP3 protein serves as a protein complex platform, while ASC bridges NLRP3 to caspase-1, allowing for its activation [30]. When caspase-1 is activated, it cleaves immature, pro-forms of interleukins (IL)-1β, IL-18, and IL-33 to mature, active forms. IL-1β, IL-18, IL-33, high mobility group box 1 protein (HMGB1), and some heat-shock proteins facilitate various inflammation processes, including fever, promoting T-cell survival, B-cell proliferation, mediation of leukocyte transmigration, etc. [33]. In addition, caspase-1 induces a pyroptosis, a special, highly inflammatory type of cell death characterized by both apoptotic and necrotic features. In this process, deoxyribonucleic acid (DNA) cleavage, nuclear condensation, and rupture of the plasma membrane are accompanied by a release of pro-inflammatory mediators including IL-1α, IL-1β, IL-18, and HMGB1 [34].

Under normal conditions, the NLRP3 complex is auto-repressed owing to an internal interaction between the NACHT domain and LRR. However, the presence of any pathogen-associated molecular patterns (PAMPs) or danger-associated molecular patterns (DAMPs) removes this auto-regression, and NLRP3 inflammasome is activated. NLRP3 acts as a general sensor of cellular stress, and several factors have been recognized as activators of NLRP3 inflammasome [31].

Crystalline silica may be also engulfed by airway epithelial cells [35] leading to activation of NLRP3 inflammasome with a subsequent release of IL-1β [36]. These findings suggest that the mechanism of NLRP3 activation is not restricted only to classical phagocytes.

Activation of NLRP3 inflammasome may be stimulated by changes in the cytosolic levels of some cations, particularly K^+^, Ca^2+^, and H^+^, while normal composition of the intracellular environment prevents the activation of NLRP3 [37,38,39]. Changes in intracellular K^+^ concentrations are strongly associated with adenosine triphosphate (ATP), while the ATP-gated ion channel triggers a rapid efflux of K^+^ from the cell. The resulting decrease in the intracellular concentration of K^+^ activates NLRP3 inflammasome and triggers ROS production [31]. Extracellular ATP released at cell death serves as an indicator of cellular damage or necrosis and thereby acts as DAMP and an activator of NLRP3. However, silica crystals may also trigger an extracellular delivery endogenous ATP [29]. Extracellular ATP [29] and ROS [40] activate a purinergic receptor, signaling via a P2X7 receptor, which mediates K^+^ efflux, maturation of IL-1, and production of additional ROS [29,41]. Moreover, P2X7 receptor allows for the influx of Na^+^ and Ca^2+^, resulting in changes to the ionic homeostasis of the cell and alteration of the second messenger signaling [42]. P2X7 also triggers a formation of pannexin 1 channels, which may carry ions and signaling molecules between the cytoplasm and the extracellular space, and thereby enables direct activation of NLRP3 by extracellular activators [43,44].

The other possibility of how the inflammasome may be activated in large particulate activators such as silica is its activation by a lysosome rupture. Release of lysosomal cysteine protease cathepsin B into the cytoplasm likely activates inflammasome directly or indirectly. Hornung et al. found that NLRP3 activation by silica crystals requires phagocytosis and that phagocytosed crystals induce lysosomal swelling and damage. NLRP3 activation was dependent on lysosomal acidification and involved cathepsin B, suggesting that crystal-induced NLRP3 inflammasome activation is more dependent on lysosomal perturbation than the crystal structure itself [45]. Nevertheless, silica surface reactivity may be also important, as the coating of quartz particles may attenuate inflammasome activation [46].

In addition, NLRP3 inflammasome is directly activated by ROS [8,31]. Whereas ROS are also generated in the previously mentioned ways of NLRP3 activation (K^+^ efflux, ATP, lysosyme rupture, silica surface) [37,47], activation by ROS seems to be a fundamental way of NLRP3 activation. However, the role of ROS in influencing the NLRP3 inflammasome might be even more complicated. Although excessive accumulation of ROS can cause cellular damage and death, generation of ROS is essential for cell signaling and several fundamental physiological responses [48]. Similarly, ROS may have contradictory roles in the activation of inflammasome [8,49].

Another way in which ROS activates the NLRP3 inflammasome is the interaction of NLRP3 with thioredoxin-interacting protein (TXNIP). In its unstimulated state, TXNIP is bound to oxidoreductase thioredoxin (TRX). Increase in intracellular ROS concentrations makes a dissociation of this complex, and TXNIP binds to NLRP3, leading to its activation [31,50]. However, it is not completely clear which primary sources of ROS are activating the inflammasome. ROS may be generated by NADPH oxidases. This way of ROS production is of high importance in silica inhalation, where ineffective clearance of phagocyted material results in chronic activation of nicotinamide adenine dinucleotide phosphate (NADPH) oxidases and excessive ROS production [47]. ROS may be also generated by the activity of other enzymes, particularly xanthine oxidase, cyclooxygenase, and lipoxygenase [48]. Another source of ROS is mitochondria, where ROS are produced in different stress conditions such as hypoxia, membrane damage, or increased metabolic rates [51]. The response of mitochondria to cellular stress is mediated through depolarization of the mitochondrial membrane, ROS release, decrease in ATP, and reversible opening of the mitochondrial permeability transition pores [52]. A subsequent increase in permeability of the mitochondria membrane may result in a release of mitochondrial contents and in eventual cell death [53]. Permeabilization of the mitochondrial membrane may be inhibited by K^+^, scavenging mitochondrial ROS, or NLRP3 deficiency, but no effect was demonstrated for cathepsin B or caspase-1 inhibitors. Contrary to this, IL-1β secretion may be suppressed by K^+^, scavenging mitochondrial ROS, and both cathepsin B and caspase-1 inhibition [52]. Mitochondrial DNA (mtDNA) released from mitochondria can activate the NLRP3 inflammasome and may thereby induce apoptosis [54]. In an attempt to avoid cellular damage, ROS-generating mitochondria are constantly removed by mitophagy, a special type of autophagy [55]. Autophagy is a cytoprotective process by which the cell isolates damaged structures and molecules such as organelles, proteins, or pathogens in a double-membrane compartment named autophagosome; targets the material for degradation in the lysosome; and reutilizes the components [56]. Inhibition of mitophagy enhances an accumulation of dysfunctional mitochondria in the cells and their breakdown, leading to an increase of mtDNA in the cytosol [57].

There is a complex interplay between ROS, inflammasome activation, and autophagy. Autophagy suppresses ROS generation [58,59] and removes intracellular mitochondrial-derived DAMPs, inflammasome components or cytokine, and may thereby inhibit inflammasome activity [60,61]. Autophagy may also negatively regulate pyroptosis [62]. Vice versa, ROS may upregulate autophagy [63], representing negative feedback to control ROS-modulated caspase-1 activation while simultaneously removing ROS-damaged organelles and proteins [60], and inflammasome negatively regulates autophagy [64]. Thus, inflammasome and autophagy may modulate each other by common inhibitory mechanisms, creating balance between both processes to maintain cell homeostasis [65].

Activation of NLRP3 inflammasome may be influenced by other bioactive agents too. NLRP3 may be activated by vimentin [66], a type III intermediate filament which is critical for stabilizing intracellular architecture [67]. On the other hand, NLRP3 inflammasome is negatively regulated by tripartite-motif protein TRIM30 via the suppression of ROS production [68]. NLRP3 activity may be also controlled by microRNA (miRNA). While miRNA-133a-1 suppresses the activation of NLRP3 and production of IL-1β by inhibiting expression of mitochondrial uncoupling protein 2 (UCP2) [69], myeloid-specific miRNA-223 suppresses NLRP3 expression via a conserved binding site within the 3’ untranslated region of NLRP3, translating into decreased NLRP3 inflammasome activity [70]. Some anti-apoptotic proteins, e.g., Bcl-2, may also inhibit inflammasome [54].

Additional tissue injury results from activation of other inflammatory pathways. A variety of DAMPs released from apoptotic or damaged cells can bind to various receptors, including TLR on macrophages, and trigger the production of cytokines and chemokines, which recruit neutrophils into the tissue [27,71]. Activation of transcriptional factors NF-κB and AP-1, leading to the production of pro-inflammatory cytokines, is also triggered by ROS [8,72].

### 2.6. Association of Persistent Silica-Induced Inflammation with Fibrosis and Autoimmune Reactions

Although the molecular mechanisms of silica-induced inflammation, fibrosis, and the related autoimmune responses have not been completely understood yet, there has been a generally accepted concept supporting the contribution of inflammation in the development of fibrosis and autoimmune responses due to silica inhalation [2,9,73,74].

Activation of the inflammatory pathways, including inflammasome and NF-κB, due to exposure to silica particles leads to increased production of pro-inflammatory cytokines. Among them, elevated levels of IL-1β, IL-18, and tumor necrosis factor (TNF)α are strongly associated with the development of lung fibrosis [75,76] as they stimulate the recruitment of fibroblasts and the proliferation of fibroblasts and mesenchymal cells to form fibroblastic foci, as well as releasing high amounts of components of the extracellular matrix, including collagen, fibronectin, hyaluronic acid, and proteoglycans [9]. IL-1β enhances the production of transforming growth factor (TGF)β, the most potent profibrotic substance, which triggers the activation, proliferation, and transdifferentiation of epithelial cells and fibroblasts into myofibroblasts generating collagen [77,78]. In addition, IL-1β stimulates the secretion of neutrophil-attracting CXC chemokines, provoking an influx of neutrophils and sparking damage to the epithelial cells, and the platelet-derived growth factor (PDGF), which promotes growth, chemotaxis, and fibrosis [79]. TGFβ, IL-1β, TNFα and ROS increase the expression of the plasminogen activator inhibitor (PAI)-1, which decreases neutrophil apoptosis and degradation of the extracellular matrix, prompts recruitment of additional inflammatory cells, and suppresses the release of anti-fibrogenic growth factors [80,81]. High production of profibrotic substances and recruitment of collagen- and fibronectin-producing cells, then, leads to the formation of silicotic nodules, scaring of the tissue, and reduction of areas for gas exchange. Due to the defect in an apoptotic cell clearance (or efferocytosis), a key process in the resolution of inflammation, silica-induced cellular death is a source of autoantigenic material, leading to the generation of autoantibodies and an autoimmune response [1,73,82].

However, there are several recent articles indicating that inhalation of micro- and nanoparticles is linked to immunosuppression [74,83]. This hypothesis is based on a finding of increased levels of TGFβ and IL-10, which have both profibrotic and immunosuppressive effects [84,85]. It is assumed that immunosuppression is initially established to limit early inflammation but later contributes to chronic fibrosis [83]. On the course of silica-mediated changes, all three types of inflammation contribute [74]. Initial reactions to inhaled silica belong to type 1 inflammation with active phagocytosis by M1 macrophages and recruitment of neutrophils, both of which are charged by Th1 lymphocytes and innate lymphoid cells (ILC) group 1, secreting interferon (IFN)-γ, IL-2, and lymphotoxin-α. Acute inflammation is followed by resolution and tissue repair mediated via specialized pro-resolving mediators and type 2 cytokines (IL-4, IL-5, IL-9, IL-10, and IL-13) and cells (M2 macrophages, ILC2 cells and Th2 lymphocytes) [74]. These cytokines recruit and activate type 2 effector cells including eosinophils, basophils, mast cells, and myofibroblasts. As silica particles cannot be digested by phagocytes, the resolution is extended and incomplete and type 2 inflammation becomes magnified, which leads to interstitial fibrosis, granuloma formation, and tumorigenesis [74]. Type 3 immune response is mediated by Th17 lymphocytes, which represent a subtype of pro-inflammatory helper CD4^+^ T-lymphocytes producing IL-17, IL-21, and IL-22 which recruit neutrophils and induce epithelial antimicrobial responses but also mediate autoimmune diseases. However, polarization of Th17 cells and overproduction of Th17 cytokines was also found in response to inhaled particles. In addition, inhalation of silica was associated with elevated regulatory T cell (Treg)-mediated immunosuppressive response, which downregulates induction and proliferation of effector T cells [86]. Thus, the silica-induced proliferation of fibroblasts and synthesis of the extracellular matrix, leading to lung fibrosis, is presumably the result of complex interactions between inflammation and immunosuppression, which need to be further elucidated [83].

## 3. Novel Treatment Possibilities

Therapeutic interventions for lung fibrotic diseases including lung silicosis are quite limited. However, recent studies have suggested the therapeutical potential of several approaches (Table 1).

### 3.1. Anti-Fibrotic Drugs

For idiopathic pulmonary fibrosis, two drugs (pirfenidone and nintedanib) have been recently approved by the U.S. Food and Drug Administration (FDA), but no disease-modifying drug has been approved for silicosis yet [87]. However, some improvement for pirfenidone and nintedanib might also be found in silicosis. In in vitro measurement, pirfenidone added to human bronchial epithelial cells suppressed silica-induced epithelial-mesenchymal transition (EMT) and activation of NLRP3 inflammasome [88]. In rats, pirfenidone reduced silica-induced inflammation and alveolar damage; decreased levels of TNFα, IL-1β and IL-6 in the lung tissue; decreased the expression of collagen I and vimentin; and suppressed the expression of TGFβ1, Smad2/3 and EMT [89]. Administration of tyrosine kinase inhibitor nintedanib in silica- and bleomycin-induced murine models of lung fibrosis inhibited PDGF receptor activation, fibroblast proliferation, and fibroblast-to-myofibroblast transformation; reduced lymphocytes and neutrophils in the bronchoalveolar lavage fluid (BALF); and decreased levels of IL-1β, keratinocyte chemoattractant (KC), tissue inhibitor of metalloproteinase-1 (TIMP-1), and collagen. Histological investigation showed diminished lung inflammation, granuloma formation, and fibrosis. The therapeutic effect was dependent on treatment onset and duration [90]. Although nintedanib is standardly given orally, inhalation was also tested in silica-exposed animals to promote the local effect of the drug. The authors have demonstrated that small-dose inhalation might be comparable with oral treatment [91].

### 3.2. Anti-Cytokine Therapy

There have been several promising approaches that might be efficient in silicosis, e.g., monoclonal antibodies against pro-inflammatory cytokines or antagonists of cytokine receptors. Due to the importance of IL-1β-driven inflammation in the development of lung silicosis, treatment with an IL-1 receptor antagonist (IL-1ra), e.g., anakinra, may reduce the proportion of damaged and fibrotic lungs. In silica- and bleomycin-instilled murine models of fibrosis, IL-1ra prevented collagen deposition, decreased the proportion of damaged lungs and silica-induced formation of nodules but had little or no influence on the number of cells in BALF [92]. In another study, anakinra and the anti-IL-17 antibody have been tested in silica-instilled mice. The monoclonal IL-17 antibody attenuated lung inflammation and accumulation of inflammatory cells, while anakinra decreased silica-induced lung inflammation and the Th17 response, indicating that IL-1β promotes inflammation by initiating a Th17 response via an IL-1β/IL-1RI-dependent mechanism [93]. The role of IL-17A and Th17 cells in the progress of silica-induced inflammation and fibrosis was confirmed by Chen et al., who found that IL-17A neutralization by the IL-17A antibody delayed neutrophil accumulation and suppressed Th17 cell development by decreasing IL-6 and/or IL-1β, increased Treg cells in an early phase of silica-induced inflammation, and delayed silica-induced Th1/Th2 immune and autoimmune responses [94]. Nevertheless, only one clinical report of anakinra use on a patient with silicosis has been published. Anakinra, given to the 37-year-old man in a dose of 100 mg/day subcutaneously for 6 months, improved his respiratory functions, oxygen saturation, and inflammatory markers (C-reactive protein, erythrocyte sedimentation rate) [95].

The blockade of IL-9, a cytokine which belongs to Th2 cytokines, might also be of benefit. In silica-challenged mice, treatment with an anti-IL-9-neutralizing antibody inhibited lung fibrosis, as assessed by lung hydroxyproline level, and suppressed the levels of cytokines and chemokines IL-1β, IL-6, IL-12, CCL2, CXCL1, and TNFα in BALF [97,98].

Participation of IL-13 in fibrogenic processes as a part of the remodeling process suggests the role of IL-13 in pulmonary fibrosis and consequently of its antagonists in treatment [139]. Delivery of a recombinant immunotoxin, comprised of human IL-13 and a mutated form of Pseudomonas exotoxin (IL-13-PE), to silica-injured mice inhibited silica-induced granuloma and fibrotic responses, while silica-induced upregulation of TNFα, TGFβ, and chemokines increased collagen deposition and airway hyperreactivity to methacholine and responded to IL-13-PE. In addition, IL-13-PE suppressed both IL-13-induced proliferation of cultured lung fibroblasts from silicotic mice and silica-induced IL-8 generation from A549 cells [99].

TNFα could be the other important target for silicosis treatment. In silica- and bleomycin-induced mice models of pulmonary fibrosis, infusion of a human recombinant soluble TNF receptor (rsTNFR-β), which acts as a TNFα antagonist, decreased biochemical markers of lung fibrosis (contents of hydroxyproline and collagen), reduced the proportion of areas in the damaged lung and, in the silicosis model, diminished the formation of nodules with rich collagen contents [100]. Administration of infliximab, a monoclonal immunoglobin G neutralizing TNFα biological activity, to rats with silica-induced acute lung injury significantly improved lung pathological changes, decreased the count of inflammatory cells and collagen deposition, declined TNFα in serum and BALF, and suppressed NF-κB signaling and expression of inducible NOS [102]. Similarly, pretreatment with infliximab in bleomycin-instilled rats effectively prevented them from inflammation, oxidative stress, and lung fibrosis [101]. Etanercept, a recombinant soluble human TNF receptor, which binds to TNF and neutralizes its activity, was subcutaneously administered to 88 patients with idiopathic pulmonary fibrosis (IPF) at a dose of 25 mg twice weekly as a sole treatment of IPF (clinical trial No. NCT 00063869). Although etanercept was well tolerated, at 48 weeks no significant differences in efficacy endpoints were observed between the groups, and a nonsignificant reduction in disease progression was seen in several physiologic, functional, and quality-of-life endpoints [140].

In silica- and bleomycin-induced experimental models of pulmonary fibrosis, treatment with monoclonal antibodies specific for the leukocytic integrins CD-11a or CD-11b prevented collagen deposition, even when given in established pulmonary fibrosis. Anti-CD-11 antibodies mitigated fibrosing alveolitis and decreased lymphoid infiltration and platelet microthrombi associated with both types of alveolitis but had little to no effect on the cellularity of BALF [103].

Due to their significant role in the progression of silica-induced fibrosis, blockade of IL-10 or intervention with IL-10 signaling pathways could be also valuable [85,141,142]. However, there has been no study published yet on the use of an IL-10 blocker in animal or clinical studies of silicosis. Additional studies are necessary to determine whether this therapeutic option might be useful for patients affected by particle-associated disorders [83].

### 3.3. Blockers of Inflammasome Activation

As demonstrated in an excellent review by Zahid et al. [143], inflammasome activity may be suppressed by a number of inhibitors acting at various levels. Primarily, inflammasome activation may be prevented by suppression of the factors leading to its activation, i.e., blocking NF-κB expression, mitochondrial stress, lysosomal damage, extracellular ATP, K^+^ efflux, etc. Oxidized ATP as an antagonist of ATP declined inflammasome activation, caspase-1-mediated cleavage of IL-1β, and K^+^ efflux in hyperoxia-exposed alveolar macrophages [144]. Suppression of activation of NLRP3 inflammasome with a subsequent reduction in IL-1β responses was observed after inhibition of phagocytosis, phagosomal acidification or cathepsin B activity [45,145], or via inhibition of K^+^ efflux by high extracellular K^+^ concentration [37,146].

In addition, there is a variety of small-molecule agents acting as indirect or direct inhibitors of inflammasome activation, inhibitors of constituents of NLRP3 inflammasome, direct inhibitors of NLRP3 protein, or caspase-1 inhibitors [143,145,147,148]. Many of these inhibitors of NLRP3 inflammasome have remarkable therapeutic potential. However, none of them has been approved by FDA, and therefore the evolution of potential inflammasome inhibitors with high specificity, stability, and low toxicity still continues [143,148].

### 3.4. Agents Enhancing an Autophagy-Lysosomal System

Crystalline silica-induced destruction of lysosomes impairs autophagic substrate degradation in alveolar macrophages. As accumulation of autophagosomes causes damage to macrophages, restoring the function of the autophagy-lysosomal system and enhancing the autophagic flux could alleviate silica-induced persistent inflammation and fibrosis [108].

Various inhibitors of lysosomal processing, which, e.g., increase lysosomal pH (lysosomotropic weak base ammonium chloride), inhibit cathepsin D (pepstatin A) or acidic sphingomyelinase (despiramine), or blunt the surface-active sites of silica crystals (aluminium lactate), may reduce activation of caspase and cell apoptosis [28]. Phagolysosome membrane permeabilization may be also blocked by imipramine, which is an FDA-approved tricyclic antidepressant drug with lysosomotropic characteristics. Imipramine decreased silica-induced cytotoxicity and release of mature IL-1β from alveolar macrophages in vitro, as well as reducing silica-induced inflammation in a short-term murine model and silica-induced lung injury and fibrosis in a long-term in vivo model [104].

As previously mentioned, autophagy is an intracellular process regulating the cycle of synthesis and degradation of cellular components. Engulfment of silica results in a lysosomal rupture, which may cause an accumulation of autophagosomes in the alveolar macrophages. The abundant accumulation of autophagosomes may lead to apoptosis in alveolar macrophages. Thus, autophagy may alleviate silica-induced pulmonary fibrosis by decreasing apoptosis of macrophages, and, vice versa, the blockade of autophagy may aggravate the apoptosis of alveolar macrophages [149,150].

Several possibilities of how to influence the autophagy activity have been recently reviewed [150,151]. For instance, the autophagy activity in fibroblasts may be increased by upregulated expression of miR326 [126], and alveolar macrophage-specific autophagy is stimulated by dioscin [103]. Enhancement of autophagy by rapamycin and cAMP protected alveolar epithelial cells from apoptosis and attenuated silica-induced pulmonary fibrosis in mice [106]. Autophagy in macrophages can be also promoted by dioscin, a component of traditional Chinese medicine, which stimulates the process of autophagic degradation, further protecting alveolar macrophages from mitochondria-dependent apoptosis [103]. Accelerated activity of autophagy and decreased apoptosis of alveolar macrophages were also observed for another natural extract, a component of Atractylodes rhizome atractylenolide III (ATL-III) [107].

Another substance influencing autophagy is trehalose, a non-reducing disaccharide, which acts as a regulator of transcription factor EB (TFEB), a master gene for lysosomal biogenesis driving expression of autophagy and lysosomal genes [152]. In silica-exposed mice, silica increased TFEB nuclear localization and expression in macrophages, while TFEB overexpression or treatment with trehalose acting as a TFEB activator alleviated lysosomal dysfunction, enhanced autophagic flux, and reduced apoptosis, inflammatory cytokine levels, and fibrosis [108]. Similarly, in alveolar macrophages collected from workers professionally exposed to silica, the addition of trehalose restored autophagy-lysosomal function, accelerated the process of autophagic degradation, and decreased levels of cleaved caspase-3 [149].

Autophagy may also be influenced by administration of mesenchymal cells (MSCs). However, as recently reviewed by Ceccariglia et al., the relation of autophagy and MSCs is complex, and the mechanisms through which MSCs can modulate the autophagy of target cells and how autophagy can affect MSCs’ therapeutic properties have been under research. Modulation of autophagy in MSCs may affect their properties and change their therapeutic potential. On the other hand, MSCs can affect autophagy in the immune cells involved in injury-induced inflammation, reducing their survival, proliferation, and function and favoring the resolution of inflammation [153]. One of the studies where the relation of autophagy and effects of MSCs were tested is the study by Zhu et al. Bone-marrow-derived MSCs decreased expressions of autophagy-associated proteins, microtubule-associated protein light chain 3 (LC-3) and autophagy-related gene Beclin-1 in alveolar macrophages and attenuated immune and autophagic activity, which may inhibit the progress of silicosis caused by damage of alveolar macrophages and promote a fibrotic lung recovery in a rat model of silicosis [133].

### 3.5. Antioxidants

Several NADPH oxidases associated with phagocytosis of silica have a common P22PHOX subunit. Blocking it may impair NLRP3 activation and thereby may be used as a therapeutic target [47]. Another promising target may be NAD-dependent deacetylase sirtuin (SIRT)-1, which stimulates the expression of antioxidants through the FoxO pathway and suppresses NF-κB signaling and NLRP3 activity. Vice versa, high concentrations of ROS can inhibit the activity of the SIRT-1 enzyme, e.g., by evoking oxidative modifications on its cysteine residues, which enhances NF-κB signaling and inflammatory responses [154].

In a rat model of silica-induced lung injury, treatment with N-acetylcysteine (NAC) reduced the fibrotic score, decreased hydroxyproline and malondialdehyde (MDA) concentrations in the lung, and prevented silica-induced increases in TNFα, IL-8 and high-sensitivity C-reactive protein in BALF and serum, as well as reducing the mitochondrial apoptosis pathway [109,110]. Similarly in silica-damaged mice, NAC ameliorated silica-induced pulmonary inflammation, lung injury, as demonstrated by down-regulation of oxidising enzymes and lower levels of MDA, enhanced antioxidant activity, increased expression of E-cadherin, and decreased expressions of vimentin and cytochrome C [111].

In recent clinical studies carried out on patients with silicosis, NAC was combined with a calcium channel blocker tetrandrine, which can also alleviate pulmonary fibrosis and inflammation by reducing the level of type I and III collagen mRNA and collagen deposition in the lung. This treatment combination improved the pulmonary function and exercise tolerance of patients and inhibited levels of IL-6, TNFα, TGFβ1, and matrix metalloproteinase (MMP)-7 [155,156].

### 3.6. Corticosteroids

Similarly, for acute lung injury [157], opinions on the use of corticosteroids in silicosis are contradictory. Several studies showed that corticosteroids may effectively mitigate acute silica-induced pulmonary inflammation. For instance, in silica-instilled rats, pretreatment with dexamethasone significantly reduced markers of inflammation and oxidative stress [112], reduced NF-κB expression, and decreased the count of total cells and neutrophils in BALF [113].

However, the efficacy of corticosteroids in the mitigation of lung fibrotic changes was largely non-homogenous and depended on the animal species or type of corticosteroid used. Barbarin et al. found that dexamethasone treatment reduced the lung fibrotic reaction in silica-injured rats, while no effect on fibrosis was found in mice [114]. Similarly, in another study on mice, dexamethasone treatment diminished lung injury, cellular inflammation and pro-inflammatory cytokine expression (TNFα, IL-1β, and KC) but had no effect on silica-induced lung fibrosis and expression of the fibrogenic and suppressive cytokines TGFβ and IL-10 [115]. In contrast, intranasal corticosteroid flunisolide inhibited granuloma and fibrotic responses, observed 28 days after the silica challenge in mice. Flunisolide suppressed the silica-induced upregulation of macrophage inflammatory proteins (MIP)-1α/CCL-3 and MIP-2/CXCL-2, decreased TNFα and TGFβ, and reduced deposition of collagen and airway hyperreactivity to methacholine. In addition, flunisolide effectively repressed the responses of proliferation and MCP-1/CCL-2 production in IL-13-stimulated lung fibroblasts from silica- or saline-challenged mice [116].

Contrary to the non-convincing effect of exogenous corticosteroids, positive results were found for endogenous glucocorticoids, i.e., for endogenous glucocorticoid-regulated protein annexin A1. Treatment with N-terminal ANXA1-derived peptide annexin 1-(2-26) in silica-instilled mice reduced concentrations of fibrotic and chemotactic cytokines, which resulted in inhibition of leukocyte migration, generation of pro-inflammatory cytokines, collagen deposition, and granuloma formation in the lung parenchyma, while these variables were only partially inhibited by dexamethasone [117].

### 3.7. Other Agents Reducing Inflammation and Fibrosis

There is a wide number of agents belonging to various pharmacological groups that may effectively suppress inflammation and fibrosis. For instance, rupatadine, a dual antagonist of histamine and platelet-activation factor (PAF), enhanced the resolution of inflammation and fibrosis in a dose-dependent manner in both bleomycin- and silica-induced pulmonary fibrosis models. Rupatadine reduced the inflammation score, collagen deposition and EMT, and infiltration or expression of inflammatory cells or cytokines in the lung tissue improved lung function and decreased animal death, while providing superior therapeutic efficacy compared with pirfenidone [96]. Nonsteroidal anti-inflammatory drug (NSAID) cyclooxygenase inhibitor piroxicam reduced lung inflammation but had no effect on the content of collagen or levels of TGFβ or IL-10 in the lungs of mice with silicosis [115]. Nicorandil, an antianginal and K^+^ channel opener agent, exerted potent antioxidant, anti-inflammatory and antifibrotic properties, which decreased cell counts, lactate dehydrogenase (LDH), TNFα, TGFβ and total protein levels and upregulated nuclear factor erythroid 2-related factor 2 (Nrf-2) and heme oxygenase-1 levels in BALF, as well as decreasing markers of inflammation (NF-κB, inducible NOS, and myeloperoxidase) in the lung, mitigating oxidative and nitrosative stress, reducing pulmonary edema and collagen deposition, and alleviating histopathological signs of pulmonary fibrosis in a rat model of silicosis [123]. Similarly, favorable results for nicorandil have been also demonstrated in rats with a bleomycin-induced model of lung fibrosis [124].

Other promising group of agents positively influencing inflammation and fibrosis are phytomedicines [158]. There is a number of natural compounds that have been successfully tested in the experimental silicosis. For instance, steroidal saponin dioscin reduced infiltration of macrophages, as well as B- and T- lymphocytes, into the lung, caused a secretion of pro-inflammatory and profibrotic cytokines, and inhibited TGFβ/Smad3 signaling and fibroblast activation in crystalline silica-induced pulmonary fibrosis in mice [159]. Dioscin can also promote autophagy in macrophages, as demonstrated in silica-injured mice, where it reduced mitochondria-dependent apoptosis and cytokine production in alveolar macrophages [105]. The natural flavonoid compound hesperetin (HSP) decreased the extent of alveolitis and pulmonary fibrosis; reduced levels of MDA; increased the activities of antioxidant enzymes and total antioxidant capacity; inhibited the synthesis and secretion of TGFβ1; reduced pro-inflammatory cytokines IL-1β, IL-4, and TNFα; and increased the levels of anti-inflammatory factors IFN-γ and IL-10 in rats exposed to silica [125]. Similar effects of hesperetin, probably mediated via inhibition of NF-κB, were also observed in a rat model of ventilator-induced acute lung injury [160].

### 3.8. Agents Increasing cAMP and cGMP

Other promising approaches include increasing concentrations of cyclic adenosine monophosphate (cAMP) and/or cyclic guanosine monophosphate (cGMP). For instance, concentrations of cAMP might be elevated by forskolin, a natural extract from Coleus forskohlii (Plectranthus barbatus), via activation of adenylyl cyclase [161]. cAMP then binds to the NLRP3 protein and targets it for degradation, thus interrupting the action of inflammasome and production of IL-1β in human macrophages [162]. However, forskolin may also down-regulate the protein levels of IL-1β and TNFα by influencing the TLR4/MyD88/NF-κB signal pathway [163].

A similar effect on cAMP and/or cGMP may be reached by phosphodiesterase (PDE) inhibitors [164]. Both nonselective and selective PDE inhibitors [118,165,166,167] may suppress tissue remodeling and lung fibrosis, and their positive effect may also be observed in silica-induced lung injury and fibrosis. For example, administration of tadalafil, a selective PDE-5 inhibitor, significantly reduced markers of inflammation (number of inflammatory cells in BALF, TNFα), lung injury (total lung protein, serum LDH activity), and oxidative stress (MDA, nitrite/nitrate), as well as increasing lung antioxidant activity (lung SOD activity and glutathione content) but decreasing TGFβ1 and collagen contents, which correlated with a decline in thickness of the blood vessel walls, restoration of normal respiratory functions and reduction in airway hyperactivity [119]. In a murine model of silicosis, another PDE-5 inhibitor sildenafil also mitigates lung inflammation without modifying collagen, TGFβ or IL-10 lung content [115].

### 3.9. Agents Influencing TGFβ-Signaling

TGFβ1 plays a key role in fibrotic tissue responses, including lung fibrosis, and the intervention of TGFβ1 expression. Its signaling appears to be highly promising therapeutic target for intervention in fibroproliferative diseases [78,168]. A variety of agents have been developed to block expression of TGFβ1, its receptor or signaling molecules at different levels of TGFβ activation and signaling, such as integrins, MMP, semaphorin 7a, phosphatase and tensin homolog agonist, prostaglandin E2, microRNA, lysyl oxidase-like 2, soluble TGFβ type II receptor (TβRII) fragments, TGFβ-neutralizing antibodies and TGFβ type I receptor (TβRI) kinase inhibitors 2, etc. [168,169]. Some of these possibilities have been successfully tested in bleomycin-induced animal models of fibrosis. For instance, transfection of the sTGFRII gene attenuated apoptosis, injury, and fibrosis [170], as well as anthraquinone emodin obtained from Rheum palmatum L., which is known for its anti-inflammatory, immunosuppressive, and pro-apoptotic effects, suppressed TGFβ-induced EMT and fibroblast activation [120,121]. Similarly, ponatinib, a multi-targeted tyrosine-kinase inhibitor, reversed EMT, inhibited apoptosis of alveolar type I cells, and prevented a pulmonary fibrosis by suppressing the TGFβ1/Smad3 pathway [122]. Another promising target may be caveolin 1, the main protein present in small invaginations of the plasma membrane of some cells (caveolae). Caveolin 1 interacts with many receptors and molecules and participates in the internalization, trafficking and degradation of TGFβ receptors [171,172]. However, because of the important function of TGFβ1 in immune and cellular homeostasis, direct or complete blocking of TGFβ1 or its signaling would not be tolerable; therefore, alternative ways of selective inhibition of the pathologic effect of TGFβ1, preserving other necessary biological functions, should be tested [168].

### 3.10. MicroRNA

Noncoding microRNAs modulate the expression of a number of protein-coding genes at a postranscriptional level, which may be associated with the development of pulmonary fibrosis [173]. As previously mentioned, several microRNA (miR-133a-1 and miR-223) suppress NLRP3 inflammasome [69,70] and therefore may be useful in the treatment of silicosis. Recently, several miRNA have been successfully used in in vitro or in vivo silica studies. For example, miR-326 inhibited inflammation and promoted autophagy activity [126], miR-449a reduced lung fibrotic lesions and upregulated autophagic activity [127], miR-326 inhibited TGFβ1 expression and attenuated the lung fibrotic response [128], miR-29b and miR-34a suppressed silica-induced EMT [129,130], miR-503 mitigated the TGFβ1-induced effects in fibroblasts [131], and miR-542-5p reduced the proliferation of fibroblasts and inhibited silica-induced pulmonary fibrosis [132].

### 3.11. Mesenchymal Stem Cells

Mesenchymal stem cells (MSCs) are multipotent cells obtained from bone marrow, adipose tissue, the umbilical cord, etc., which serve as progenitors for connective tissue cells and stimulate the growth, repair, and survival of other cells and tissues [174]. Due to their beneficial immunomodulatory and regenerative capacity, MSCs are a potential therapy for several acute and chronic lung diseases, including pulmonary fibrosis [175,176]. Within the last decade, there have been several clinical studies on the use of MSCs in IPF [177,178,179], showing a good safety profile.

However, less is known about the possible advantages of stem cell therapy in silicosis. In several experimental studies, this kind of therapy exerted valuable effects on inflammation and lung fibrosis. For instance, the use of adipose-derived MSCs in silica-induced lung injury in rats suppressed the inflammatory response and expression of caspase-3 protein and increased the Bcl-2/Bax ratio, suggesting anti-fibrotic effects [134]. Treatment with bone marrow mesenchymal stem/stromal cells enhanced the expression of epithelial marker proteins and decreased the expression of fibrosis marker proteins, while attenuating the Wnt/β-catenin signaling pathway, which is abnormally activated in silica-induced pulmonary fibrosis [135]. Similarly, injection of bone marrow MSCs-conditioned medium attenuated silica-induced pulmonary fibrosis; decreased the collagen deposition and number of nodules; and suppressed collagen I, collagen III, and fibronectin mRNA, as well as the content of TGFβ1 and hydroxyproline, while alveolar epithelial markers were upregulated [136]. As mentioned before, transplantation of mesenchymal cells may also influence the autophagic activity of alveolar macrophages [133].

Treatment with autologous bone marrow MSCs was used in four patients with pulmonary silicosis who had developed lung fibrosis (clinical trial No. NCT01977131). The patients were given autologous bone marrow MSCs previously transfected by a vector containing human hepatocyte growth factor (HGF) cDNA (MSCs/HGF), which were intravenously administered weekly for three consecutive weeks at a dose of 2 × 10^6^ cells/kg [180]. The treatment was safe, and the respiratory symptoms (cough and chest distress) ameliorated within six months post-therapy, accompanied by an improvement in pulmonary function. The ratios of peripheral CD4- and CD8-positive cell concentrations elevated, serum IgG levels returned to their normal value, and a computer tomography investigation showed partial absorption of the nodular and reticulonodular lesions in the lung during following twelve months [180]. Another small prospective, non-randomized, single-center longitudinal study was carried out on five patients with silicosis. Bone marrow-derived mononuclear cells (BMDMCs) were administered via bronchoscopy (2 × 10^7^ cells) into both lung lobes. Similar to the previously mentioned study, no adverse events were observed during or after BMDMC administration. Lung function, quality of life, and radiologic features remained stable throughout the follow-up, while an early increase in perfusion in the lung base was observed and sustained after BMDMC administration (clinical trial No. NCT01239862) [181].

Although either infusion or instillation of mesenchymal stem stromal or progenitor cells have been well tolerated almost without serious adverse events causally related to cell treatment [182], there have been some safety concerns raised about the risk of the undesirable differentiation of transplanted MSCs resulting in possible malignant transformation and vascular occlusion [174,183]. For these reasons, several studies have evaluated the properties of soluble factors and extracellular vesicles of MSCs secretome, which can be a safer alternative to MSCs since they are cell-free and have a better immunogenicity, tumorigenicity, and embolism formation side effect profile than MSCs. In addition, they can mimic many of the desired clinical effects of MSCs [184,185,186]. Extracellular vesicles can be classified into three groups according to size, i.e., exosomes, microvesicles/ectosomes, and apoptotic bodies [174]. Administration of various extracellular vesicles was successfully tested in animal models of silicosis that decreased the influx of inflammatory cells, collagen deposition, enhanced mitochondria bioenergetics, and suppressed macrophage activation [137,138].

## 4. Conclusions

Development of silica-induced inflammation and fibrosis is a result of complex interactions between several pro-inflammatory and profibrotic factors. Elucidation of the key role of NLRP3 inflammasome; NLRP3 inflammasome-associated changes, particularly cleavage of IL-1β and activation of caspase-1; and identification of factors contributing to NLRP3 activation have revealed several potential targets for the therapy. Many of these approaches have been successfully tested in experimental models of silicosis, and some of them have been used in small clinical studies. In conclusion, better understanding of pathophysiology has opened up new perspectives for targeted treatment of silicosis; however, further evaluation in randomized clinical studies is needed.

## Figures and Tables

**Table 1:** Treatment possibilities tested in animal models of silicosis or fibrosis.

Treatment	References
Anti-fibrotic drugs	
Pirfenidone	[89,96]
Nintedanib	[90,91]
Anti-cytokine therapy	
Anakinra (IL-1ra)	[92,93]
Anti-IL-17 antibody	[93,94]
Anti-IL-9 antibody	[97,98]
IL-13 immunotoxin	[99]
Recombinant soluble TNF receptor	[100]
Infliximab	[101,102]
Anti CD-11 antibodies	[103]
Agents influencing autophagy-lysosomal system	
Imipramine	[104]
Dioscin	[105]
Rapamycin/cAMP	[106]
Atractylenolide III	[107]
Trehalose	[108]
Antioxidants	
N-acetylcysteine	[109,110,111]
Corticosteroids	
Dexamethasone	[112,113,114,115]
Flunisolide	[116]
Endogenous glucocorticoids	
Annexin A1	[117]
Agents increasing cAMP	
Roflumilast	[118]
Tadalafil	[119]
Sildenafil	[115]
Agents influencing TGFβ	
Emodin	[120,121]
Ponatinib	[122]
Other agents	
Rupatadine	[96]
Piroxicam	[115]
Nicorandil	[123,124]
Hesperetin	[125]
MicroRNA	[126,127,128,129,130,131,132]
Mesenchymal cells	[133,134,135,136]
Extracellular vesicles	[137,138]

## References

[1] Pollard K.M. (2016). Silica, silicosis, and autoimmunity. Front. Immunol..

[2] Barnes H., Goh N.S.L., Leong T.L., Hoy R. (2019). Silica-associated lung disease: An old-world exposure in modern industries. Respirology.

[3] Greenberg M.I., Waksman J., Curtis J. (2007). Silicosis: A review. Dis. Mon..

[4] Talini D., Paggiaro P.L., Falaschi F., Battolla L., Carrara M., Petrozzino M., Begliomini E., Bartolozzi C., Giuntini C. (1995). Chest radiography and high resolution computed tomography in the evaluation of workers exposed to silica dust: Relation with functional findings. Occup. Environ. Med..

[5] Antao V.C., Pinheiro G.A., Terra-Filho M., Kavakama J., Müller N.L. (2005). High-resolution CT in silicosis: Correlation with radiographic findings and functional impairment. J. Comput. Assist. Tomogr..

[6] Leung C.C., Yu I.T., Chen W. (2012). Silicosis. Lancet.

[7] Hamilton R.F., Thakur S.A., Holian A. (2008). Silica binding and toxicity in alveolar macrophages. Free Radic. Biol. Med..

[8] Harijith A., Ebenezer D.L., Natarajan V. (2014). Reactive oxygen species at the crossroads of inflammasome and inflammation. Front. Physiol..

[9] Sayan M., Mossman B.T. (2016). The NLRP3 inflammasome in pathogenic particle and fibre-associated lung inflammation and diseases. Part. Fibre Toxicol..

[10] Palecanda A., Kobzik L. (2001). Receptors for unopsonized particles: The role of alveolar macrophage scavenger receptors. Curr. Mol. Med..

[11] Iyer R., Hamilton R.F., Li L., Holian A. (1996). Silica-induced apoptosis mediated via scavenger receptor in human alveolar macrophages. Toxicol. Appl. Pharmacol..

[12] Tsuda A., Henry F.S., Butler J.P. (2013). Particle transport and deposition: Basic physics of particle kinetics. Compr. Physiol..

[13] Bowden D.H., Hedgecock C., Adamson I.Y. (1989). Silica-induced pulmonary fibrosis involves the reaction of particles with interstitial rather than alveolar macrophages. J. Pathol..

[14] Lapp N.L., Castranova V. (1993). How silicosis and coal workers’ pneumoconiosis develop-a cellular assessment. Occup. Med..

[15] Vallyathan V., Shi X.L., Dalal N.S., Irr W., Castranova V. (1988). Generation of free radicals from freshly fractured silica dust. Potential role in acute silica-induced lung injury. Am. Rev. Respir. Dis..

[16] Castranova V. (2004). Signaling pathways controlling the production of inflammatory mediators in response to crystalline silica exposure: Role of reactive oxygen/nitrogen species. Free Radic. Biol. Med..

[17] Kanj R.S., Kang J.L., Castranova V. (2006). Interaction between primary alveolar macrophages and primary alveolar type II cells under basal conditions and after lipopolysaccharide or quartz exposure. J. Toxicol. Environ. Health A.

[18] Liu X., Keane M.J., Harrison J.C., Cilento E.V., Ong T., Wallace W.E. (1998). Phospholipid surfactant adsorption by respirable quartz and in vitro expression of cytotoxicity and DNA damage. Toxicol. Lett..

[19] Spech R.W., Wisniowski P., Kachel D.L., Wright J.R., Martin W.J. (2000). Surfactant protein A prevents silica-mediated toxicity to rat alveolar macrophages. Am. J. Physiol. Lung Cell. Mol. Physiol..

[20] Yildirim B.B., Akgedik R., Akgedik S., Nazaroglu H. (2016). Pulmonary alveolar proteinosis in a marble worker. Int. J. Occup. Med. Environ. Health..

[21] Vallyathan V., Kang J.H., Van Dyke K., Dalal N.S., Castranova V. (1991). Response of alveolar macrophages to in vitro exposure to freshly fractured versus aged silica dust: The ability of Prosil 28, an organosilane material, to coat silica and reduce its biological reactivity. J. Toxicol. Environ. Health.

[22] Vallyathan V., Castranova V., Pack D., Leonard S., Shumaker J., Hubbs A.F., Shoemaker D.A., Ramsey D.M., Pretty J.R., McLaurin J.L. (1995). Freshly fractured quartz inhalation leads to enhanced lung injury and inflammation. Potential role of free radicals. Am. J. Respir. Crit. Care Med..

[23] Castranova V., Vallyathan V., Ramsey D.M., McLaurin J.L., Pack D., Leonard S., Barger M.W., Ma J.Y., Dalal N.S., Teass A. (1997). Augmentation of pulmonary reactions to quartz inhalation by trace amounts of iron-containing particles. Environ. Health Perspect..

[24] Fubini B., Giamello E., Volante M., Bolis V. (1990). Chemical functionalities at the silica surface determining its reactivity when inhaled. Formation and reactivity of surface radicals. Toxicol. Ind. Health.

[25] Castranova V. (1994). Generation of oxygen radicals and mechanisms of injury prevention. Environ. Health Perspect..

[26] Janssen Y.M., Marsh J.P., Absher M.P., Hemenway D., Vacek P.M., Leslie K.O., Borm P.J., Mossman B.T. (1992). Expression of antioxidant enzymes in rat lungs after inhalation of asbestos or silica. J. Biol. Chem..

[27] Øvrevik J., Refsnes M., Låg M., Holme J.A., Schwarze P.E. (2015). Activation of proinflammatory responses in cells of the airway mucosa by particulate matter: Oxidant- and non-oxidant-mediated triggering mechanisms. Biomolecules.

[28] Thibodeau M.S., Giardina C., Knecht D.A., Helble J., Hubbard A.K. (2004). Silica-induced apoptosis in mouse alveolar macrophages is initiated by lysosomal enzyme activity. Toxicol. Sci..

[29] Riteau N., Baron L., Villeret B., Guillou N., Savigny F., Ryffel B., Rassendren F., Le Bert M., Gombault A., Couillin I. (2012). ATP release and purinergic signaling: A common pathway for particle-mediated inflammasome activation. Cell Death Dis..

[30] Hoffman H.M., Wanderer A.A. (2010). Inflammasome and IL-1beta-mediated disorders. Curr. Allergy Asthma Rep..

[31] Tschopp J., Schroder K. (2010). NLRP3 inflammasome activation: The convergence of multiple signalling pathways on ROS production?. Nat. Rev. Immunol..

[32] Barbé F., Douglas T., Saleh M. (2014). Advances in Nod-like receptors (NLR) biology. Cytokine Growth Factor Rev..

[33] Sims J.E., Smith D.E. (2010). The IL-1 family: Regulators of immunity. Nat. Rev. Immunol..

[34] Bergsbaken T., Fink S.L., Cookson B.T. (2009). Pyroptosis: Host cell death and inflammation. Nat. Rev. Microbiol..

[35] Øvrevik J., Refsnes M., Namork E., Becher R., Sandnes D., Schwarze P.E., Låg M. (2006). Mechanisms of silica-induced IL-8 release from A549 cells: Initial kinase-activation does not require EGFR activation or particle uptake. Toxicology.

[36] Peeters P.M., Perkins T.N., Wouters E.F., Mossman B.T., Reynaert N.L. (2013). Silica induces NLRP3 inflammasome activation in human lung epithelial cells. Part. Fibre Toxicol..

[37] Pétrilli V., Papin S., Dostert C., Mayor A., Martinon F., Tschopp J. (2007). Activation of the NALP3 inflammasome is triggered by low intracellular potassium concentration. Cell Death Differ..

[38] Ichinohe T., Pang I.K., Iwasaki A. (2010). Influenza virus activates inflammasomes via its intracellular M2 ion channel. Nat. Immunol..

[39] Lee G.S., Subramanian N., Kim A.I., Aksentijevich I., Goldbach-Mansky R., Sacks D.B., Germain R.N., Kastner D.L., Chae J.J. (2012). The calcium-sensing receptor regulates the NLRP3 inflammasome through Ca^2+^ and cAMP. Nature.

[40] Solini A., Menini S., Rossi C., Ricci C., Santini E., Blasetti Fantauzzi C., Iacobini C., Pugliese G. (2013). The purinergic 2X7 receptor participates in renal inflammation and injury induced by high-fat diet: Possible role of NLRP3 inflammasome activation. J. Pathol..

[41] Luna-Gomes T., Santana P.T., Coutinho-Silva R. (2015). Silica-induced inflammasome activation in macrophages: Role of ATP and P2X7 receptor. Immunobiology.

[42] Coutinho-Silva R., Persechini P.M. (1997). P2Z purinoceptor-associated pores induced by extracellular ATP in macrophages and J774 cells. Am. J. Physiol..

[43] Ferrari D., Pizzirani C., Adinolfi E., Lemoli R.M., Curti A., Idzko M., Panther E., Di Virgilio F. (2006). The P2X7 receptor: A key player in IL-1 processing and release. J. Immunol..

[44] Kanneganti T.-D., Lamkanfi M., Nunez G. (2007). Intracellular NOD-like receptors in host defenseand disease. Immunity.

[45] Hornung V., Bauernfeind F., Halle A., Samstad E.O., Kono H., Rock K.L., Fitzgerald K.A., Latz E. (2008). Silica crystals and aluminum salts activate the NALP3 inflammasome through phagosomal destabilization. Nat. Immunol..

[46] Peeters P.M., Eurlings I.M., Perkins T.N., Wouters E.F., Schins R.P., Borm P.J., Drommer W., Reynaert N.L., Albrecht C. (2014). Silica-induced NLRP3 inflammasome activation in vitro and in rat lungs. Part. Fibre Toxicol..

[47] Dostert C., Pétrilli V., Van Bruggen R., Steele C., Mossman B.T., Tschopp J. (2008). Innate immune activation through Nalp3 inflammasome sensing of asbestos and silica. Science.

[48] Knaus U.G. (2021). Oxidants in physiological processes. Handb. Exp. Pharmacol..

[49] Meissner F., Molawi K., Zychlinsky A. (2008). Superoxide dismutase 1 regulates caspase-1 and endotoxic shock. Nat. Immunol..

[50] Zhou R., Tardivel A., Thorens B., Choi I., Tschopp J. (2010). Thioredoxin-interacting protein links oxidative stress to inflammasome activation. Nat. Immunol..

[51] Brookes P.S., Yoon Y., Robotham J.L., Anders M.W., Sheu S.-S. (2004). Calcium, ATP, and ROS: A mitochondrial love-hate triangle. Am. J. Physiol. Cell Physiol..

[52] Heid M.E., Keyel P.A., Kamga C., Shiva S., Watkins S.C., Salter R.D. (2013). Mitochondrial reactive oxygen species induces NLRP3-dependent lysosomal damage and inflammasome activation. J. Immunol..

[53] Green D.R., Kroemer G. (2004). The pathophysiology of mitochondrial cell death. Science.

[54] Shimada K., Crother T.R., Karlin J., Dagvadorj J., Chiba N., Chen S., Ramanujan V.K., Wolf A.J., Vergnes L., Ojcius D.M. (2012). Oxidized mitochondrial DNA activates the NLRP3 inflammasome during apoptosis. Immunity.

[55] Goldman S.J., Taylor R., Zhang Y., Jin S. (2010). Autophagy and the degradation of mitochondria. Mitochondrion.

[56] Deretic V., Levine B. (2009). Autophagy, immunity, and microbial adaptations. Cell Host Microbe.

[57] Nakahira K., Haspel J.A., Rathinam V.A., Lee S.J., Dolinay T., Lam H.C., Englert J.A., Rabinovitch M., Cernadas M., Kim H.P. (2011). Autophagy proteins regulate innate immune responses by inhibiting the release of mitochondrial DNA mediated by the NALP3 inflammasome. Nat. Immunol..

[58] Bensaad K., Cheung E.C., Vousden K.H. (2009). Modulation of intracellular ROS levels by TIGAR controls autophagy. EMBO J..

[59] Rouschop K.M., Ramaekers C.H., Schaaf M.B., Keulers T.G., Savelkouls K.G., Lambin P., Koritzinsky M., Wouters B.G. (2009). Autophagy is required during cycling hypoxia to lower production of reactive oxygen species. Radiother. Oncol..

[60] Schroder K., Tschopp J. (2010). The inflammasomes. Cell.

[61] Sun Q., Fan J., Billiar T.R., Scott M.J. (2017). Inflammasome and autophagy regulation-a two-way street. Mol. Med..

[62] Suzuki T., Núñez G. (2008). A role for Nod-like receptors in autophagy induced by Shigella infection. Autophagy.

[63] Huang J., Brumell J.H. (2009). NADPH oxidases contribute to autophagy regulation. Autophagy.

[64] Yuk J.M., Jo E.K. (2013). Crosstalk between autophagy and inflammasomes. Mol. Cells.

[65] de Lavera I., Pavon A.D., Paz M.V., Oropesa-Avila M., de la Mata M., Alcocer-Gomez E., Garrido-Maraver J., Cotan D., Alvarez-Cordoba M., Sanchez-Alcazar J.A. (2017). The connections among autophagy, inflammasome and mitochondria. Curr. Drug Targets.

[66] dos Santos G., Rogel M.R., Baker M.A., Troken J.R., Urich D., Morales-Nebreda L., Sennello J.A., Kutuzov M.A., Sitikov A., Davis J.M. (2015). Vimentin regulates activation of the NLRP3 inflammasome. Nat. Commun..

[67] Eriksson J.E., Dechat T., Grin B., Helfand B., Mendez M., Pallari H.M., Goldman R.D. (2009). Introducing intermediate filaments: From discovery to disease. J. Clin. Investig..

[68] Hu Y., Mao K., Zeng Y., Chen S., Tao Z., Yang C., Sun S., Wu X., Meng G., Sun B. (2010). Tripartite-motif protein 30 negatively regulates NLRP3 inflammasome activation by modulating reactive oxygen species production. J. Immunol..

[69] Bandyopadhyay S., Lane T., Venugopal R., Parthasarathy P.T., Cho Y., Galam L., Lockey R., Kolliputi N. (2013). MicroRNA-133a-1 regulates inflammasome activation through uncoupling protein-2. Biochem. Biophys. Res. Commun..

[70] Bauernfeind F., Rieger A., Schildberg F.A., Knolle P.A., Schmid-Burgk J.L., Hornung V. (2012). NLRP3 inflammasome activity is negatively controlled by miR-223. J. Immunol..

[71] Rock K.L., Kono H. (2008). The inflammatory response to cell death. Annu. Rev. Pathol..

[72] Porter D.W., Ye J., Ma J., Barger M., Robinson V.A., Ramsey D., McLaurin J., Khan A., Landsittel D., Teass A. (2002). Time course of pulmonary response of rats to inhalation of crystalline silica: NF-kappa B activation, inflammation, cytokine production, and damage. Inhal. Toxicol..

[73] Pollard K.M. (2020). Perspective: The lung, particles, fibers, nanomaterials, and autoimmunity. Front. Immunol..

[74] Ma Q. (2020). Polarization of immune cells in the pathologic response to inhaled particulates. Front. Immunol..

[75] Davis G.S., Pfeiffer L.M., Hemenway D.R. (1998). Persistent overexpression of interleukin-1beta and tumor necrosis factor-alpha in murine silicosis. J. Environ. Pathol. Toxicol. Oncol..

[76] Biswas R., Bunderson-Schelvan M., Holian A. (2011). Potential role of the inflammasome-derived inflammatory cytokines in pulmonary fibrosis. Pulm. Med..

[77] dos Santos G., Kutuzov M.A., Ridge K.M. (2012). The inflammasome in lung diseases. Am. J. Physiol. Lung Cell Mol. Physiol..

[78] Pardali E., Sanchez-Duffhues G., Gomez-Puerto M.C., Ten Dijke P. (2017). TGF-β-induced endothelial-mesenchymal transition in fibrotic diseases. Int. J. Mol. Sci..

[79] Wynn T.A. (2008). Cellular and molecular mechanisms of fibrosis. J. Pathol..

[80] Liu R.M. (2008). Oxidative stress, plasminogen activator inhibitor 1, and lung fibrosis. Antioxid. Redox Signal.

[81] Zmijewski J.W., Bae H.B., Deshane J.S., Peterson C.B., Chaplin D.D., Abraham E. (2011). Inhibition of neutrophil apoptosis by PAI-1. Am. J. Physiol. Lung Cell. Mol. Physiol..

[82] Lescoat A., Ballerie A., Lelong M., Augagneur Y., Morzadec C., Jouneau S., Jégo P., Fardel O., Vernhet L., Lecureur V. (2020). Crystalline silica impairs efferocytosis abilities of human and mouse macrophages: Implication for silica-associated systemic sclerosis. Front. Immunol..

[83] Huaux F. (2018). Emerging role of immunosuppression in diseases induced by micro- and nano-particles: Time to revisit the exclusive inflammatory scenario. Front. Immunol..

[84] Jagirdar J., Begin R., Dufresne A., Goswami S., Lee T.C., Rom W.N. (1996). Transforming growth factor-beta (TGF-beta) in silicosis. Am. J. Respir. Crit. Care Med..

[85] Sun L., Louie M.C., Vannella K.M., Wilke C.A., LeVine A.M., Moore B.B., Shanley T.P. (2011). New concepts of IL-10-induced lung fibrosis: Fibrocyte recruitment and M2 activation in a CCL2/CCR2 axis. Am. J. Physiol. Lung Cell. Mol. Physiol..

[86] Annunziato F., Romagnani C., Romagnani S. (2015). The 3 major types of innate and adaptive cell-mediated effector immunity. J. Allergy Clin. Immunol..

[87] Rosenbloom J., Macarak E., Piera-Velazquez S., Jimenez S.A. (2017). Human fibrotic diseases: Current challenges in fibrosis research. Methods Mol. Biol..

[88] Li X., Yan X., Wang Y., Wang J., Zhou F., Wang H., Xie W., Kong H. (2018). NLRP3 inflammasome inhibition attenuates silica-induced epithelial to mesenchymal transition (EMT) in human bronchial epithelial cells. Exp. Cell Res..

[89] Guo J., Yang Z., Jia Q., Bo C., Shao H., Zhang Z. (2019). Pirfenidone inhibits epithelial-mesenchymal transition and pulmonary fibrosis in the rat silicosis model. Toxicol. Lett..

[90] Wollin L., Maillet I., Quesniaux V., Holweg A., Ryffel B. (2014). Antifibrotic and anti-inflammatory activity of the tyrosine kinase inhibitor nintedanib in experimental models of lung fibrosis. J. Pharmacol. Exp. Ther..

[91] Epstein-Shochet G., Pham S., Beck S., Naiel S., Mekhael O., Revill S., Hayat A., Vierhout M., Bardestein-Wald B., Shitrit D. (2020). Inhalation: A means to explore and optimize nintedanib’s pharmacokinetic/pharmacodynamic relationship. Pulm. Pharmacol. Ther..

[92] Piguet P.F., Vesin C., Grau G.E., Thompson R.C. (1993). Interleukin 1 receptor antagonist (IL-1ra) prevents or cures pulmonary fibrosis elicited in mice by bleomycin or silica. Cytokine.

[93] Song L., Weng D., Dai W., Tang W., Chen S., Li C., Chen Y., Liu F., Chen J. (2014). Th17 can regulate silica-induced lung inflammation through an IL-1beta-dependent mechanism. J. Cell. Mol. Med..

[94] Chen Y., Li C., Weng D., Song L., Tang W., Dai W., Yu Y., Liu F., Zhao M., Lu C. (2014). Neutralization of interleukin-17A delays progression of silica-induced lung inflammation and fibrosis in C57BL/6 mice. Toxicol. Appl. Pharmacol..

[95] Cavalli G., Fallanca F., Dinarello C.A., Dagna L. (2015). Treating pulmonary silicosis by blocking interleukin 1. Am. J. Respir. Crit. Care Med..

[96] Lv X.X., Wang X.X., Li K., Wang Z.Y., Li Z., Lv Q., Fu X.M., Hu Z.W. (2013). Rupatadine protects against pulmonary fibrosis by attenuating PAF-mediated senescence in rodents. PLoS ONE.

[97] Sugimoto N., Suzukawa M., Nagase H., Koizumi Y., Ro S., Kobayashi K., Yoshihara H., Kojima Y., Kamiyama-Hara A., Hebisawa A. (2019). IL-9 blockade suppresses silica-induced lung inflammation and fibrosis in mice. Am. J. Respir. Cell Mol. Biol..

[98] Rojas-Quintero J., Wang X., Owen C.A. (2019). Dusting off IL-9 as a new therapeutic target for pulmonary fibrosis. Am. J. Respir. Cell. Mol. Biol..

[99] Ferreira T.P., de Arantes A.C., do Nascimento C.V., Olsen P.C., Trentin P.G., Rocco P.R., Hogaboam C.M., Puri R.K., Martins M.A., Silva P.M. (2013). IL-13 immunotoxin accelerates resolution of lung pathological changes triggered by silica particles in mice. J. Immunol..

[100] Piguet P.F., Vesin C. (1994). Treatment by human recombinant soluble TNF receptor of pulmonary fibrosis induced by bleomycin or silica in mice. Eur. Respir. J..

[101] Altintas N., Erboga M., Aktas C., Bilir B., Aydin M., Sengul A., Ates Z., Topcu B., Gurel A. (2016). Protective effect of infliximab, a tumor necrosis factor-alfa inhibitor, on bleomycin-induced lung fibrosis in rats. Inflammation.

[102] Zhang H., Sui J.N., Gao L., Guo J. (2018). Subcutaneous administration of infliximab-attenuated silica-induced lung fibrosis. Int. J. Occup. Med. Environ. Health.

[103] Piguet P.F., Rosen H., Vesin C., Grau G.E. (1993). Effective treatment of the pulmonary fibrosis elicited in mice by bleomycin or silica with anti-CD-11 antibodies. Am. Rev. Respir. Dis..

[104] Biswas R., Trout K.L., Jessop F., Harkema J.R., Holian A. (2017). Imipramine blocks acute silicosis in a mouse model. Part. Fibre Toxicol..

[105] Du S., Li C., Lu Y., Lei X., Zhang Y., Li S., Liu F., Chen Y., Weng D., Chen J. (2019). Dioscin alleviates crystalline silica-induced pulmonary inflammation and fibrosis through promoting alveolar macrophage autophagy. Theranostics.

[106] Zhao X., Wei S., Li Z., Lin C., Zhu Z., Sun D., Bai R., Qian J., Gao X., Chen G. (2019). Autophagic flux blockage in alveolar epithelial cells is essential in silica nanoparticle-induced pulmonary fibrosis. Cell Death Dis..

[107] Chen S., Tang K., Hu P., Tan S., Yang S., Yang C., Chen G., Luo Y., Zou H. (2021). Atractylenolide III alleviates the apoptosis through inhibition of autophagy by the mTOR-dependent pathway in alveolar macrophages of human silicosis. Mol. Cell. Biochem..

[108] He X., Chen S., Li C., Ban J., Wei Y., He Y., Liu F., Chen Y., Chen J. (2020). Trehalose alleviates crystalline silica-induced pulmonary fibrosis via activation of the TFEB-mediated autophagy-lysosomal system in alveolar macrophages. Cells.

[109] Zhang H., Yin G., Jiang H., Zhang C. (2013). High-dose N-acetylcysteine decreases silica-induced lung fibrosis in the rat. J. Int. Med. Res..

[110] Zhang L., He Y.L., Li Q.Z., Hao X.H., Zhang Z.F., Yuan J.X., Bai Y.P., Jin Y.L., Liu N., Chen G. (2014). N-acetylcysteine alleviated silica-induced lung fibrosis in rats by down-regulation of ROS and mitochondrial apoptosis signaling. Toxicol. Mech. Methods.

[111] Huang H., Chen M., Liu F., Wu H., Wang J., Chen J., Liu M., Li X. (2019). N-acetylcysteine therapeutically protects against pulmonary fibrosis in a mouse model of silicosis. Biosci. Rep..

[112] Van Dyke K., Antonini J.M., Wu L., Ye Z., Reasor M.J. (1994). The inhibition of silica-induced lung inflammation by dexamethasone as measured by bronchoalveolar lavage fluid parameters and peroxynitrite-dependent chemiluminescence. Agents Actions.

[113] Sacks M., Gordon J., Bylander J., Porter D., Shi X.L., Castranova V., Kaczmarczyk W., Van Dyke K., Reasor M.J. (1998). Silica-induced pulmonary inflammation in rats: Activation of NF-kappa B and its suppression by dexamethasone. Biochem. Biophys. Res. Commun..

[114] Barbarin V., Nihoul A., Misson P., Arras M., Delos M., Leclercq I., Lison D., Huaux F. (2005). The role of pro- and anti-inflammatory responses in silica-induced lung fibrosis. Respir. Res..

[115] Rabolli V., Lo Re S., Uwambayinema F., Yakoub Y., Lison D., Huaux F. (2011). Lung fibrosis induced by crystalline silica particles is uncoupled from lung inflammation in NMRI mice. Toxicol. Lett..

[116] Ferreira T.P.T., Lima J.G.M.E., Farias-Filho F.A., Jannini de Sá Y.A.P., de Arantes A.C.S., Guimarães F.V., Carvalho V.F., Hogaboam C., Wallace J., Martins M.A. (2020). Intranasal flunisolide suppresses pathological alterations caused by silica particles in the lungs of mice. Front. Endocrinol..

[117] Trentin P.G., Ferreira T.P., Arantes A.C., Ciambarella B.T., Cordeiro R.S., Flower R.J., Perretti M., Martins M.A., Silva P.M. (2015). Annexin A1 mimetic peptide controls the inflammatory and fibrotic effects of silica particles in mice. Br. J. Pharmacol..

[118] Sisson T.H., Christensen P.J., Muraki Y., Dils A.J., Chibucos L., Subbotina N., Tohyama K., Horowitz J.C., Matsuo T., Bailie M. (2018). Phosphodiesterase 4 inhibition reduces lung fibrosis following targeted type II alveolar epithelial cell injury. Physiol. Rep..

[119] Abdelaziz R.R., Elkashef W.F., Said E. (2016). Tadalafil reduces airway hyperactivity and protects against lung and respiratory airways dysfunction in a rat model of silicosis. Int. Immunopharmacol..

[120] Chen X.H., Sun R.S., Hu J.M., Mo Z.Y., Yang Z.F., Jin G.Y., Guan W.D., Zhong N.S. (2009). Inhibitory effect of emodin on bleomycin-induced pulmonary fibrosis in mice. Clin. Exp. Pharmacol. Physiol..

[121] Guan R., Wang X., Zhao X., Song N., Zhu J., Wang J., Wang J., Xia C., Chen Y., Zhu D. (2016). Emodin ameliorates bleomycin-induced pulmonary fibrosis in rats by suppressing epithelial-mesenchymal transition and fibroblast activation. Sci. Rep..

[122] Qu Y., Zhang L., Kang Z., Jiang W., Lv C. (2015). Ponatinib ameliorates pulmonary fibrosis by suppressing TGF-β1/Smad3 pathway. Pulm. Pharmacol. Ther..

[123] El-Kashef D.H. (2018). Nicorandil ameliorates pulmonary inflammation and fibrosis in a rat model of silicosis. Int. Immunopharmacol..

[124] Kseibati M.O., Shehatou G.S.G., Sharawy M.H., Eladl A.E., Salem H.A. (2020). Nicorandil ameliorates bleomycin-induced pulmonary fibrosis in rats through modulating eNOS, iNOS, TXNIP and HIF-1α levels. Life Sci..

[125] Li S., Shao L., Fang J., Zhang J., Chen Y., Yeo A.J., Lavin M.F., Yu G., Shao H. (2021). Hesperetin attenuates silica-induced lung injury by reducing oxidative damage and inflammatory response. Exp. Ther. Med..

[126] Xu T., Yan W., Wu Q., Xu Q., Yuan J., Li Y., Li P., Pan H., Ni C. (2019). MiR-326 inhibits inflammation and promotes autophagy in silica-induced pulmonary fibrosis through targeting TNFSF14 and PTBP1. Chem. Res. Toxicol..

[127] Han R., Ji X., Rong R., Li Y., Yao W., Yuan J., Wu Q., Yang J., Yan W., Han L. (2016). MiR-449a regulates autophagy to inhibit silica-induced pulmonary fibrosis through targeting Bcl2. J. Mol. Med..

[128] Das S., Kumar M., Negi V., Pattnaik B., Prakash Y.S., Agrawal A., Ghosh B. (2014). MicroRNA-326 regulates profibrotic functions of transforming growth factor-β in pulmonary fibrosis. Am. J. Respir. Cell Mol. Biol..

[129] Sun J., Li Q., Lian X., Zhu Z., Chen X., Pei W., Li S., Abbas A., Wang Y., Tian L. (2019). MicroRNA-29b mediates lung mesenchymal-epithelial transition and prevents lung fibrosis in the silicosis model. Mol. Ther. Nucleic Acids.

[130] Qi Y., Zhao A., Yang P., Jin L., Hao C. (2020). miR-34a-5p Attenuates EMT through targeting SMAD4 in silica-induced pulmonary fibrosis. J. Cell Mol. Med..

[131] Wu Q., Han L., Gui W., Wang F., Yan W., Jiang H. (2020). MiR-503 suppresses fibroblast activation and myofibroblast differentiation by targeting VEGFA and FGFR1 in silica-induced pulmonary fibrosis. J. Cell. Mol. Med..

[132] Yuan J., Li P., Pan H., Li Y., Xu Q., Xu T., Ji X., Liu Y., Yao W., Han L. (2018). miR-542-5p attenuates fibroblast activation by targeting integrin α6 in silica-induced pulmonary fibrosis. Int. J. Mol. Sci..

[133] Zhu H.X., Gao J.L., Zhao M.M., Li R., Tian Y.X., Wang X., Zhang J., Yuan J.X., Cui J.Z. (2016). Effects of bone marrow-derived mesenchymal stem cells on the autophagic activity of alveolar macrophages in a rat model of silicosis. Exp. Ther. Med..

[134] Chen S., Cui G., Peng C., Lavin M.F., Sun X., Zhang E., Yang Y., Guan Y., Du Z., Shao H. (2018). Transplantation of adipose-derived mesenchymal stem cells attenuates pulmonary fibrosis of silicosis via anti-inflammatory and anti-apoptosis effects in rats. Stem Cell Res. Ther..

[135] Zhang E., Yang Y., Chen S., Peng C., Lavin M.F., Yeo A.J., Li C., Liu X., Guan Y., Du X. (2018). Bone marrow mesenchymal stromal cells attenuate silica-induced pulmonary fibrosis potentially by attenuating Wnt/β-catenin signaling in rats. Stem Cell Res. Ther..

[136] Li X., An G., Wang Y., Liang D., Zhu Z., Tian L. (2018). Targeted migration of bone marrow mesenchymal stem cells inhibits silica-induced pulmonary fibrosis in rats. Stem Cell Res. Ther..

[137] Choi M., Ban T., Rhim T. (2014). Therapeutic use of stem cell transplantation for cell replacement or cytoprotective effect of microvesicle released from mesenchymal stem cell. Mol. Cells.

[138] Bandeira E., Oliveira H., Silva J.D., Menna-Barreto R.F.S., Takyia C.M., Suk J.S., Witwer K.W., Paulaitis M.E., Hanes J., Rocco P.R.M. (2018). Therapeutic effects of adipose-tissue-derived mesenchymal stromal cells and their extracellular vesicles in experimental silicosis. Respir. Res..

[139] Passalacqua G., Mincarini M., Colombo D., Troisi G., Ferrari M., Bagnasco D., Balbi F., Riccio A., Canonica G.W. (2017). IL-13 and idiopathic pulmonary fibrosis: Possible links and new therapeutic strategies. Pulm. Pharmacol. Ther..

[140] Raghu G., Brown K.K., Costabel U., Cottin V., du Bois R.M., Lasky J.A., Thomeer M., Utz J.P., Khandker R.K., McDermott L. (2008). Treatment of idiopathic pulmonary fibrosis with etanercept: An exploratory, placebo-controlled trial. Am. J. Respir. Crit. Care Med..

[141] Sziksz E., Pap D., Lippai R., Béres N.J., Fekete A., Szabó A.J., Vannay Á. (2015). Fibrosis related inflammatory mediators: Role of the IL-10 cytokine family. Mediat. Inflamm..

[142] Steen E.H., Wang X., Balaji S., Butte M.J., Bollyky P.L., Keswani S.G. (2020). The role of the anti-inflammatory cytokine interleukin-10 in tissue fibrosis. Adv. Wound Care.

[143] Zahid A., Li B., Kombe A.J.K., Jin T., Tao J. (2019). Pharmacological inhibitors of the NLRP3 inflammasome. Front. Immunol..

[144] Kolliputi N., Shaik R.S., Waxman A.B. (2010). The inflammasome mediates hyperoxia-induced alveolar cell permeability. J. Immunol..

[145] Sandberg W.J., Låg M., Holme J.A., Friede B., Gualtieri M., Kruszewski M., Schwarze P.E., Skuland T., Refsnes M. (2012). Comparison of non-crystalline silica nanoparticles in IL-1β release from macrophages. Part. Fibre Toxicol..

[146] Martinon F., Mayor A., Tschopp J. (2009). The inflammasomes: Guardians of the body. Annu. Rev. Immunol..

[147] Saleh M., Mathison J.C., Wolinski M.K., Bensinger S.J., Fitzgerald P., Droin N., Ulevitch R.J., Green D.R., Nicholson D.W. (2006). Enhanced bacterial clearance and sepsis resistance in caspase-12-deficient mice. Nature.

[148] Caseley E.A., Poulter J.A., Rodrigues F. (2020). Immunome project consortium for autoinflammatory disorders (ImmunAID), McDermott MF. Inflammasome inhibition under physiological and pharmacological conditions. Genes Immun..

[149] Tan S., Yang S., Chen G., Zhu L., Sun Z., Chen S. (2020). Trehalose alleviates apoptosis by protecting the autophagy-lysosomal system in alveolar macrophages during human silicosis. Life Sci..

[150] Tan S., Chen S. (2021). Macrophage Autophagy and Silicosis: Current Perspective and Latest Insights. Int. J. Mol. Sci..

[151] Zhao H., Wang Y., Qiu T., Liu W., Yao P. (2020). Autophagy, an important therapeutic target for pulmonary fibrosis diseases. Clin. Chim. Acta.

[152] Settembre C., Di Malta C., Polito V.A., Garcia Arencibia M., Vetrini F., Erdin S., Erdin S.U., Huynh T., Medina D., Colella P. (2011). TFEB links autophagy to lysosomal biogenesis. Science.

[153] Ceccariglia S., Cargnoni A., Silini A.R., Parolini O. (2020). Autophagy: A potential key contributor to the therapeutic action of mesenchymal stem cells. Autophagy.

[154] Salminen A., Kaarniranta K., Kauppinen A. (2013). Crosstalk between oxidative stress and SIRT1: Impact on the aging process. Int. J. Mol. Sci..

[155] Sun J., Song P., Wang Y., Chen Y. (2019). Clinical efficacy of acetylcysteine combined with tetrandrine tablets in the treatment of silicosis and the effect on serum IL-6 and TNF-alpha. Exp. Ther. Med..

[156] Zhang J., Wang Y., Zhang S., Li J., Fang H. (2020). Effects of tetrandrine combined with acetylcysteine on exercise tolerance, pulmonary function and serum TNF-β1 and MMP-7 in silicosis patients. Exp. Ther. Med..

[157] Mokra D., Mikolka P., Kosutova P., Mokry J. (2019). Corticosteroids in acute lung injury: The dilemma continues. Int. J. Mol. Sci..

[158] Jahan S., Kumar D., Chaturvedi S., Rashid M., Wahajuddin M., Khan Y.A., Goyal S.N., Patil C.R., Mohanraj R., Subramanya S. (2017). Therapeutic targeting of NLRP3 inflammasomes by natural products and pharmaceuticals: A novel mechanistic approach for inflammatory diseases. Curr. Med. Chem..

[159] Li C., Lu Y., Du S., Li S., Zhang Y., Liu F., Chen Y., Weng D., Chen J. (2017). Dioscin exerts protective effects against crystalline silica-induced pulmonary fibrosis in mice. Theranostics.

[160] Ma H., Feng X., Ding S. (2015). Hesperetin attenuates ventilator-induced acute lung injury through inhibition of NF-κB-mediated inflammation. Eur. J. Pharmacol..

[161] Alasbahi R.H., Melzig M.F. (2012). Forskolin and derivatives as tools for studying the role of cAMP. Pharmazie.

[162] Chen Y., Wen J.G., Feng J.J., Wang Y.H., Li T.F., Nurmi K., Eklund K.K., Xing D. (2019). Forskolin attenuates the NLRP3 inflammasome activation and IL-1β secretion in human macrophages. Pediatr. Res..

[163] Du X., Shi R., Wang Y., Wu W., Sun S., Dai Z., Chen C., Weng Z., Li X., Liu Q. (2019). Isoforskolin and forskolin attenuate lipopolysaccharide-induced inflammation through TLR4/MyD88/NF-kappaB cascades in human mononuclear leukocytes. Phytother. Res..

[164] Mokra D., Mokry J. (2021). Phosphodiesterase inhibitors in acute lung injury: What are the perspectives?. Int. J. Mol. Sci..

[165] Fehrholz M., Glaser K., Speer C.P., Seidenspinner S., Ottensmeier B., Kunzmann S. (2017). Caffeine modulates glucocorticoid-induced expression of CTGF in lung epithelial cells and fibroblasts. Respir. Res..

[166] Kolb M., Raghu G., Wells A.U., Behr J., Richeldi L., Schinzel B., Quaresma M., Stowasser S., Martinez F.J. (2018). INSTAGE investigators. Nintedanib plus sildenafil in patients with idiopathic pulmonary fibrosis. N. Engl. J. Med..

[167] Wu Y., Tian Y.J., Le M.L., Zhang S.R., Zhang C., Huang M.X., Jiang M.Y., Zhang B., Luo H.B. (2020). Discovery of novel selective and orally bioavailable phosphodiesterase-1 inhibitors for the efficient treatment of idiopathic pulmonary fibrosis. J. Med. Chem..

[168] Lee C.M., Park J.W., Cho W.K., Zhou Y., Han B., Yoon P.O., Chae J., Elias J.A., Lee C.G. (2014). Modifiers of TGF-β1 effector function as novel therapeutic targets of pulmonary fibrosis. Korean J. Intern. Med..

[169] Walton K.L., Johnson K.E., Harrison C.A. (2017). Targeting TGF-β mediated SMAD signaling for the prevention of fibrosis. Front. Pharmacol..

[170] Yamada M., Kuwano K., Maeyama T., Yoshimi M., Hamada N., Fukumoto J., Egashira K., Hiasa K., Takayama K., Nakanishi Y. (2007). Gene transfer of soluble transforming growth factor type II receptor by in vivo electroporation attenuates lung injury and fibrosis. J. Clin. Pathol..

[171] Miyasato S.K., Loeffler J., Shohet R., Zhang J., Lindsey M., Le Saux C.J. (2011). Caveolin-1 modulates TGF-β1 signaling in cardiac remodeling. Matrix Biol..

[172] Gvaramia D., Blaauboer M.E., Hanemaaijer R., Everts V. (2013). Role of caveolin-1 in fibrotic diseases. Matrix Biol..

[173] Pandit K.V., Milosevic J., Kaminski N. (2011). MicroRNAs in idiopathic pulmonary fibrosis. Transl. Res..

[174] Worthington E.N., Hagood J.S. (2020). Therapeutic use of extracellular vesicles for acute and chronic lung disease. Int. J. Mol. Sci..

[175] Tzouvelekis A., Toonkel R., Karampitsakos T., Medapalli K., Ninou I., Aidinis V., Bouros D., Glassberg M.K. (2018). Mesenchymal stem cells for the treatment of idiopathic pulmonary fibrosis. Front. Med..

[176] Cruz F.F., Rocco P.R.M. (2020). The potential of mesenchymal stem cell therapy for chronic lung disease. Expert Rev. Respir. Med..

[177] Tzouvelekis A., Paspaliaris V., Koliakos G., Ntolios P., Bouros E., Oikonomou A., Zissimopoulos A., Boussios N., Dardzinski B., Gritzalis D. (2013). A prospective, non-randomized, no placebo-controlled, phase Ib clinical trial to study the safety of the adipose derived stromal cells-stromal vascular fraction in idiopathic pulmonary fibrosis. J. Transl. Med..

[178] Chambers D.C., Enever D., Ilic N., Sparks L., Whitelaw K., Ayres J., Yerkovich S.T., Khalil D., Atkinson K.M., Hopkins P.M. (2014). A phase 1b study of placenta-derived mesenchymal stromal cells in patients with idiopathic pulmonary fibrosis. Respirology.

[179] Glassberg M.K., Minkiewicz J., Toonkel R.L., Simonet E.S., Rubio G.A., DiFede D., Shafazand S., Khan A., Pujol M.V., LaRussa V.F. (2017). Allogeneic human mesenchymal stem cells in patients with idiopathic pulmonary fibrosis via intravenous delivery (AETHER): A phase I safety clinical trial. Chest.

[180] Liu W.W., Wang H.X., Yu W., Bi X.Y., Chen J.Y., Chen L.Z., Ding L., Han D.M., Guo Z.K., Lei Y.X. (2015). Treatment of silicosis with hepatocyte growth factor-modified autologous bone marrow stromal cells: A non-randomized study with follow-up. Genet. Mol. Res..

[181] Morales M.M., Souza S.A., Loivos L.P., Lima M.A., Szklo A., Vairo L., Brunswick T.H., Gutfilen B., Lopes-Pacheco M., Araújo A.J. (2015). Pilot safety study of intrabronchial instillation of bone marrow-derived mononuclear cells in patients with silicosis. BMC. Pulm. Med..

[182] Zhao R., Su Z., Wu J., Ji H.L. (2017). Serious adverse events of cell therapy for respiratory diseases: A systematic review and meta-analysis. Oncotarget.

[183] Volarevic V., Markovic B.S., Gazdic M., Volarevic A., Jovicic N., Arsenijevic N., Armstrong L., Djonov V., Lako M., Stojkovic M. (2018). Ethical and safety issues of stem cell-based therapy. Int. J. Med. Sci..

[184] Phinney D.G., Di Giuseppe M., Njah J., Sala E., Shiva S., St Croix C.M., Stolz D.B., Watkins S.C., Di Y.P., Leikauf G.D. (2015). Mesenchymal stem cells use extracellular vesicles to outsource mitophagy and shuttle microRNAs. Nat. Commun..

[185] Seo Y., Kim H.S., Hong I.S. (2019). Stem cell-derived extracellular vesicles as immunomodulatory therapeutics. Stem Cells Int..

[186] Guo H., Su Y., Deng F. (2020). Effects of mesenchymal stromal cell-derived extracellular vesicles in lung diseases: Current status and future perspectives. Stem Cell Rev. Rep..

